# Characterization, Quantification, and Molecular Identification of Co-Infection of Canine Parvovirus (CPV-2) Variants in Dogs Affected by Gastroenteritis in Ecuador During 2022–2023

**DOI:** 10.3390/vetsci12010046

**Published:** 2025-01-11

**Authors:** Anthony Loor-Giler, Silvana Santander-Parra, Sara Castillo-Reyes, Martin Campos, Renán Mena-Pérez, Santiago Prado-Chiriboga, Luis Nuñez

**Affiliations:** 1 Laboratorios de Investigación, Dirección General de Investigación, Universidad de Las Américas (UDLA), Antigua Vía a Nayón S/N, Quito EC 170124, Ecuador; a.abel.loor.giler@gmail.com; 2 Facultad de Ingeniería y Ciencias Aplicadas, Carrera de Ingeniería en Biotecnología, Universidad de Las Américas (UDLA), Antigua Vía a Nayón S/N, Quito EC 170124, Ecuador; 3 Facultad de Ciencias de La Salud, Carrera de Medicina Veterinaria, Universidad de Las Américas (UDLA), Antigua Vía a Nayón S/N, Quito EC 170124, Ecuador; silvanahsp@yahoo.com (S.S.-P.); sara.castillo.reyes@udla.edu.ec (S.C.-R.); rpmena@uce.edu.ec (R.M.-P.); sdpch2021@gmail.com (S.P.-C.); 4 Facultad de Industrias Agropecuarias y Ciencias Ambientales, Carrera de Medicina Veterinaria, Universidad Politécnica Estatal del Carchi (UPEC), Antisana S/N y Av. Universitaria, Tulcán EC 040102, Ecuador; rolando.campos@upec.edu.ec; 5 Facultad de Ciencias Veterinarias, Universidad Nacional de Rosario (UNR), Boulevard Ovidio Lagos y Ruta 33 Casilda, Santa Fe S2000, Argentina; 6 Facultad de Medicina Veterinaria y Zootecnia, Universidad Central del Ecuador, Gatto Sobral y Jerónimo Leiton, Quito EC 170521, Ecuador; 7 Clínica Veterinaria Docente, Universidad de Las Américas (UDLA), Calle Shuara N40-55y Av. De Los Granados, Quito EC 170503, Ecuador; 8 One Health Research Group, Facultad de Ciencias de la Salud, Universidad de Las Américas (UDLA), Antigua Vía a Nayón S/N, Quito EC 170124, Ecuador

**Keywords:** CPV-2, genotypes, co-infections, qPCR

## Abstract

Canine parvovirus (CPV-2) is a highly contagious virus in canines, and it is mostly spread by touching infected feces. Dogs of all ages can catch it, but puppies are more likely to suffer from it. Severe signs include vomiting, diarrhea with blood, feeling tired, and not drinking enough water. There are three different types of the original CPV-2 that have been found so far, which are CPV-2a, 2b, and 2c. The genome of CPV-2 is about 5.2 kb long and has two open reading frames (ORFs), namely the VP region and the NS region. Based on changes in amino acids at position 426, the VP2 protein distinguishes the gene apart in the VP region. Using a molecular method, this study contemplated the presence of CPV-2 and its variants in dogs that had gastroenteritis, as well as other infections. There were 511 samples tested, and 401 (78.47%) of them were positive for CPV-2. Of these, 144 (25.91%) were positive for the original genotype, 258 (64.34%) for genotype 2a, 343 (85.54%) for genotype 2b, and 167 (41.65%) for genotype 2c. Using the multiplex qPCR for genotyping, CPV-2a and CPV-2b were determined as the most frequent co-infections (16.45%). The three genotypes (2a, 2b, and 2c) were found in the samples examined based on the amino acids at position 426 of the VP2 protein, as demonstrated by the VP2 gene sequencing. Furthermore, it was discovered that in certain samples, a genetic modification at position 297 was connected to the virus’s pathogenicity.

## Simple Summary

Canine parvovirus (CPV-2) is a gastrointestinal virus that affects dogs, causing bloody diarrhea, vomiting, and dehydration, with the most severe symptoms occurring in canines. Since its emergence, three CPV-2-derived genotypes, CPV-2a, CPV-2b, and CPV-2c, have been described; these arise from mutations in the VP2 protein at residue 426, and their pathogenic effect is compounded. The present study identifies the presence of CPV-2, in 78.47% of the positive samples, with CPV-2b identified as the predominant genotype at 84.54%, using qPCR assays as a method of identification and quantitation of viral particles in infected dogs with gastroenteririts in Ecuador. The sequences of the VP2 region on the three genotypes were obtained using SANGER sequencing, and the presence of mutations in the amino acids of this protein related to the pathogenicity of genotypes 2b and 2c was identified in residue 297. The control of infectious diseases in domestic animals is necessary to safeguard the survival of these animals, so it is necessary to perform constant monitoring to determine the epidemiological status of these pathogens in order to be able to take appropriate containment measures.

## 1. Introduction

Canine parvovirus (CPV) is one of the most common causes of acute viral gastroenteritis, especially in puppies, where morbidity is 80% for unvaccinated puppies. The virus is thought to have originated as a variant of feline panleukopenia virus (FPV) that has adapted to canine hosts, thus possessing similar regions in its genome [1,2].

Canine parvovirus type 2 (CPV-2) is an important pathogen in domestic and wild canines that has spread worldwide since its emergence in 1970 [3,4]. It is part of the genus *Protoparvovirus* and the family *Parvoviridae*. CPV-2 has a linear single-stranded DNA genome of ~5.2 Kb, with two open reading frames (ORFs) [5,6]. The NS region codes for non-structural proteins (NS1 and NS2) involved in the replication processes of the viral genetic material, and the VP region codes for viral capsid proteins (VP1 and VP2) which have identical sequences, except for the unique 143 amino acid terminal ends of VP1 [6].

Gene and antigen variations have caused the structure of the VP2 protein to change very quickly. The CPV-2a variant was first described in 1978, with several alterations in the VP2 protein, specifically at positions 87, 101, and 305, utilized for genotyping [7]. Two more forms determined a mutation present in the coding region for an amino acid at position 426 of the VP2 protein (Asn for CPV-2 and CPV-2a); CPV-2b (Asp) and CPV-2c (Glu) were later described in 1984 [8] and 2000 [9], respectively. CPV-2 antigenic types are found all over the world, and their genetic profile and frequency vary depending on where they are found. Infections have been reported in Africa, Asia, Australia, the United States, and Europe [7,8].

In Ecuador, the presence of the three variants of canine parvovirus has recently been reported, which circulate in both vaccinated and unvaccinated canines. Current canine parvovirus vaccines based on the CPV-2 variant do not provide sufficient immunity to the emerging variants, so the virus has spread with voracity [9,10]. Not enough information has been provided on the behavior of canine parvovirus in cases of co-infections with several variants; it is unknown whether the mortality rate could increase or what is the frequency of distribution of combinations among the already existing variants [11].

By utilizing qPCR, this study aims to ascertain the prevalence of CPV-2 and its variants in co-infection among dogs diagnosed with gastroenteritis in Ecuador. Additionally, it is aimed to analyze the variants that are present in patients who are CPV-2-positive and sequence the samples in order to detect potential alterations in the circulating sequences in Ecuador. Considering the variations in the infection of every genotype of CPV-2, we have questioned the national distribution of the genotypes of this virus and whether the circulating vaccinations are preventing its spread.

## 2. Materials and Methods

### 2.1. Sampling

For this study, 511 fecal samples from dogs suffering from gastroenteritis were processed. The canines in the sample ranged in age (Supplementary Materials File) and displayed symptoms that were consistent with gastrointestinal disorders, such as fever, bloody stools, diarrhea, vomiting, loss of appetite, and dehydration. The veterinary hospital of the Universidad de Las Americas received the samples from various provinces of Ecuador [Carchi (65 samples), Chimborazo (4 samples), Guayas (50 samples), Imbabura (28 samples), Pichincha (298 samples), and Santo Domingo de los Tsáchilas (66 samples)] to determine the causal agent of the digestive symptoms. For this, the samples were received at the research laboratories of the Universidad de Las Americas and were subjected to a molecular analysis for the identification of CPV-2 and the determination of co-infections between the different variants of the virus among the samples and to molecularly characterize the current strains of CPV-2 circulating in Ecuador. All procedures conducted in the present investigation were in accordance with the guidelines and the approval of the Committee on the Care and Use of Laboratory and Domestic Animal resources of the Agency of Regulation and Control of Phytosanitary and Animal Health of Ecuador (AGROCALIDAD) under number #INT/DA/019.

### 2.2. DNA Extraction

A 1:1 suspension of the sample was made in a 2 mL microcentrifuge microtube with 1 mL of 1X phosphate-buffered saline (PBS) at pH 7.4. The sample was homogenized and frozen at −80 °C for 10 min, then thawed in a water bath at 56 °C for 1 min and again homogenized. This procedure was repeated three times and then the samples were centrifuged at 12,000× *g* for 20 min. A 200 µL aliquot of the supernatant was placed in a 1.5 mL microcentrifuge microtube and subjected to the extraction protocol using the phenol/chloroform DNA extraction [12]. DNA samples were diluted at 1:10 with ultrapure water before passing through the molecular assays.

### 2.3. qPCR Assay for CPV-2 Detection

In the present study, CPV-2 was detected through the use of a hydrolysis probe and a pair of primers that targeted a conserved region of the NS gene that is common in all CPV-2 genotypes (Table 1). The qPCR reaction was used with a final volume of 20 µL, 10 µL of TaqMan™ Universal Master Mix II (Applied Biosystems, Carlsbad, CA 92008, USA), 0.2 µM of each primer, 0.05 µM of the probe, 5 µL de ADN, and UltraPure™ DNase/RNase-Free Distilled Water dH2O (Invitrogen by Thermofisher Scientific, New York, NY 14072, USA) necessary to complete 25 µL. The amplification protocol was used according to the indications of the reagent in standard mode, with a 2 min cycle at 50 °C for UNG incubation, a 2 min cycle at 95 °C for initial denaturation, and 40 cycles of 95 °C for a 3 s denaturation at 56 °C for 30 s for annealing and a 72 °C extension of the DNA template.

For the standard curve construction, a CPV-2-positive sample was subjected to endpoint PCR, for amplifying the complete NS gene using the primers NS-FEXT and NS-REXT (Table 1). The PCR product was subjected to enzymatic purification using ExoSAP-IT Express (Applied Biosystems by Thermofisher Scientific, California, CA, USA) according to the manufacturer’s instructions. The DNA Copy Number and dilution Calculator web tool (Thermo Fisher Scientific, California, CA, USA) was used to calculate the quantity of recombinant DNA necessary to make the first dilution with an identified quantity of DNA copies, then tenfold dilutions from 10^9^ copies to 10^0^ copies were prepared to determine the sensitivity and amplification efficiency of the qPCR assay. Based on the calibration curve, all samples that showed at least one copy of genetic material were deemed positive.

### 2.4. Multiplex qPCR for CPV-2 Genotyping

Multiplex qPCR was performed to determine the CPV-2 genotypes present in the positive samples using 2 primer pairs (CPV-305 and 426) and 4 hydrolysis probes (CPV-2 305, CPV-2a 426, 2b 426, and 2c 426) (Table 1). These primers work simultaneously with 3 probes for the discrimination of the SNPs that determine the CPV-2 variants at positions 305 and 426 [14]. For the qPCR reaction, the reaction is prepared as described in Section 2.3, including the amplification protocol with a modification for annealing to 63 °C for 30 s. The presence of CPV-2 gene copies (GCs) of CPV-2 variants were determined using the calibration curve equation for each qPCR (>95% efficiency), grouping them on average according to the age of the canines.

### 2.5. Sequencing of VP1 Gene

Positive samples with the highest viral load (greater than 10,000 copies) quantified by a qPCR assay were selected and subjected to an endpoint PCR protocol to amplify the entire VP1 gene, using primers 2161F and 4823R (Table 1) employing the previously described protocol by Rez et al., 2014 [7,8]. The PCR product was subjected to enzymatic purification using ExoSAP-IT Express (Applied Biosystems by Thermofisher Scientific, California, CA, USA) according to the manufacturer’s instructions. The purified PCR product was used for SANGER-type sequencing in the forward and reverse directions using a BigDye^®^ Terminator v3.1 Cycle Sequencing kit (Thermo Fisher Scientific, Carlsbad, CA, USA). Sequencing reactions were performed with an ABI 3730 DNA Analyzer (Thermo Fisher Scientific, Carlsbad, CA, USA). The primer walking strategy was used to obtain the complete sequence of the VP1 gene.

### 2.6. Phylogenetic Analyses

The electropherograms obtained were analyzed and edited using the Genious Prime^®^ 2022.2.2 software; the ORF finder tool in Geneious was used for determining the complete CDs of the VP2 gene. The BLAST tool was also used to compare the similarity of each obtained sequence with others of CPV-2 present in GenBank. An alignment was built with the VP2 obtained sequences and other sequences of different strains of the CPV-2 existing in the GeneBank, both for the nucleotide (NT) and amino acid (AA) sequences using Clustal X 2.1 software. Subsequently, a phylogenetic analysis was performed using the MEGA11: Molecular Evolutionary Genetics Analysis version 11 [15]. A phylogenetic tree was generated using the neighbor-joining method, the p-distance substitution model including substitutions and transversions using Bootstrap 1000, and a chicken parvovirus VP sequence was added as an outgroup. The sequences were used to determine the similarity of NTs and AAs among them and with sequences of CPV-2 existing in GenBank that were previously reported.

### 2.7. GenBank Accession Numbers

The nucleotide sequences obtained from the VP region and identified in the present study were deposited in NCBI GenBank with the accession numbers UDLA 151D ECU (PQ133206); UDLA 223D ECU (PQ133207); UDLA 224D ECU (PQ133208): UDLA 90D ECU (PQ133209); UDLA 282D ECU (PQ133210); UDLA 246D ECU (PQ133211); UDLA 425D ECU (PQ133212); UDLA 245D ECU (PQ133213); UDLA 6D ECU (PQ133214); UDLA 195D ECU (PQ133215); UDLA 429D ECU (PQ133216); UDLA 15D ECU (PQ133217); UDLA 89D ECU (PQ133218); UDLA 95D ECU (PQ133219); UDLA 82D ECU (PQ133220); and UDLA 88D ECU (PQ133221).

### 2.8. VP2 Analysis

To analyze the changes in amino acid residues in the VP2 protein, a translation of the VP2 ORF of the sequenced samples was performed using the Geneious Prime^®^ 2022.2.2 program [16]. We aligned the pre-sequences obtained by MUSCLE 5.1. and grouped the genotypes based on the phylogenetic analysis and position 426 of the protein and selected the mutations of interest along the VP2 protein.

### 2.9. Statistical Analysis

Descriptive statistics of the results for CPV-2 detection and genotypes identified based on canine age were conducted, forming age groups according to stages of immunological development and average adulthood considerations. To identify whether the samples are approaching a normal distribution, a Shapiro–Wilk test was applied. The presence of CPV-2 and its variants were related to the age of the patients from which the samples used in this study were taken. A one-way ANOVA was performed on the positive data for every CPV-2 genotype to determine if the presence of viruses was related to the age of the patients, and a chi-squared test was carried out to determine whether there are significant differences in the occurrence of CPV-2 between the provinces studied and according to every age group created. All analyses used a significance level of 5% and were performed using Jaimovi 2.3.24.

## 3. Results

### 3.1. CPV-2 Detection and Genotyping

The standard curve was used to quantify CPV-2, showing an efficiency of 99.9%, with a limit of quantification (LoQ) and limit of detection (LoD) of one copy of the viral genetic material. The 511 samples were submitted to the qPCR assay for CPV-2 detection and were quantified using absolute quantification. Of the analyzed samples with the qPCR assay, in 401 samples, DNA of CPV-2 was detected (Table 2). The group with the highest number of positive samples for CPV-2 and with all variants was the group of canines between 3 and 12 months of age. The group with the lowest number of positive samples for CPV-2 and all variants were dogs at 132 to 180 months of age. The original CPV-2 variant was the least detected in the positive samples. A decline in the quantity of positive samples is observed as age increases, with the age group decreasing from 13 months to 180 months (Table 2). The present study showed that the presence of CPV-2, CPV-2b, and CPV-2c variants were related to each other according to age (*p*-value of <0.001), whereas the CPV-2a variant is not linked to the others based on age (Table 2).

In the analysis of CPV-2 genotypes using qPCR, a total of 144 positives samples was found for the original CPV-2 genotype, 258 for the CPV-2a genotype, 343 for the CPV-2b genotype, and 167 for the CPV-2c genotype (Table 2), showing that genotype 2b is the most present genotype and the original genotype CPV-2 is the least present in samples. Analysis of genotypes by the qPCR assay showed different combinations in 401 positive analyzed samples. The most frequent combination was combination C.9 (Table 3), which includes CPV-2a and CPV-2b. Individually, the variants of CPV-2 showed 2, 39, 70, and 17 positive samples for the genotypes CPV-2, 2a, 2b, and 2c, respectively, with genotype b being the most predominant individually, with 45.8% more positive samples compared to the other genotypes; 39 samples showed co-infection with all genotypes (combination C1). All other combinations included at least one individual case (Table 3).

### 3.2. Quantification of CPV-2 Variants

**Figure 1:**
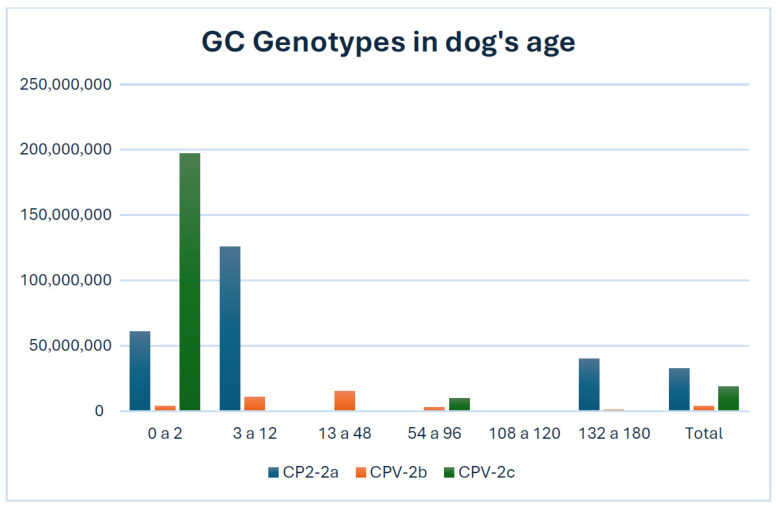
Analysis of CPV-2 variants according to the age of the patients provided using the GC (gene copy) average of genotypes 2a, 2b, and 2c for more graphical information. CPV-2 was not included due to non-significant genome copies at any age.

In determining the viral load of each genotype, it was found that on average, the genotypes with the highest viral charge in all samples were CPV-2a and 2c in the total samples (Figure 1).

Groups up to 2 months and up to 96 months had a higher CPV-2c genotype viral load, while groups up to 12 months and up to 132 months had a higher CPV-2a genotype viral load and only the group up to 96 had a higher CPV-2b genotype viral load on average. Some single samples showed viral loads exceeding one billion copies, with samples of genotype 2a and 2c showing the highest viral load (Table 4). Only the original genotype CPV-2 showed low viral loads across all groups, with the highest recorded viral load being 1612 in the group aged 0 to 2 months (Table 4).

### 3.3. Analysis by Location

According to the analysis of positive samples collected in six provinces of Ecuador, the province with the highest percentage of positive samples is Pichincha, then Santo Domingo de los Tsachillas, Guayas, Imbabura, and those with the lowest percentage are Carchi and Chimborazo (Table 5). CPV-2 was detected in all provinces from which samples were collected, notwithstanding the relatively small sample sizes for each province. Based on the statistical analysis, it was determined that provinces do indeed influence the precedence of CPV-2; nevertheless, it is important to consider that over 50% of all samples collected in Pichincha were acquired as a bias factor, considering that the country’s capital is located in this province, which is one of the most populous and the site where the study was conducted; the study population was centralized in this region.

### 3.4. Sequencing of VP1 and Phylogenetic Analysis

**Figure 2:**
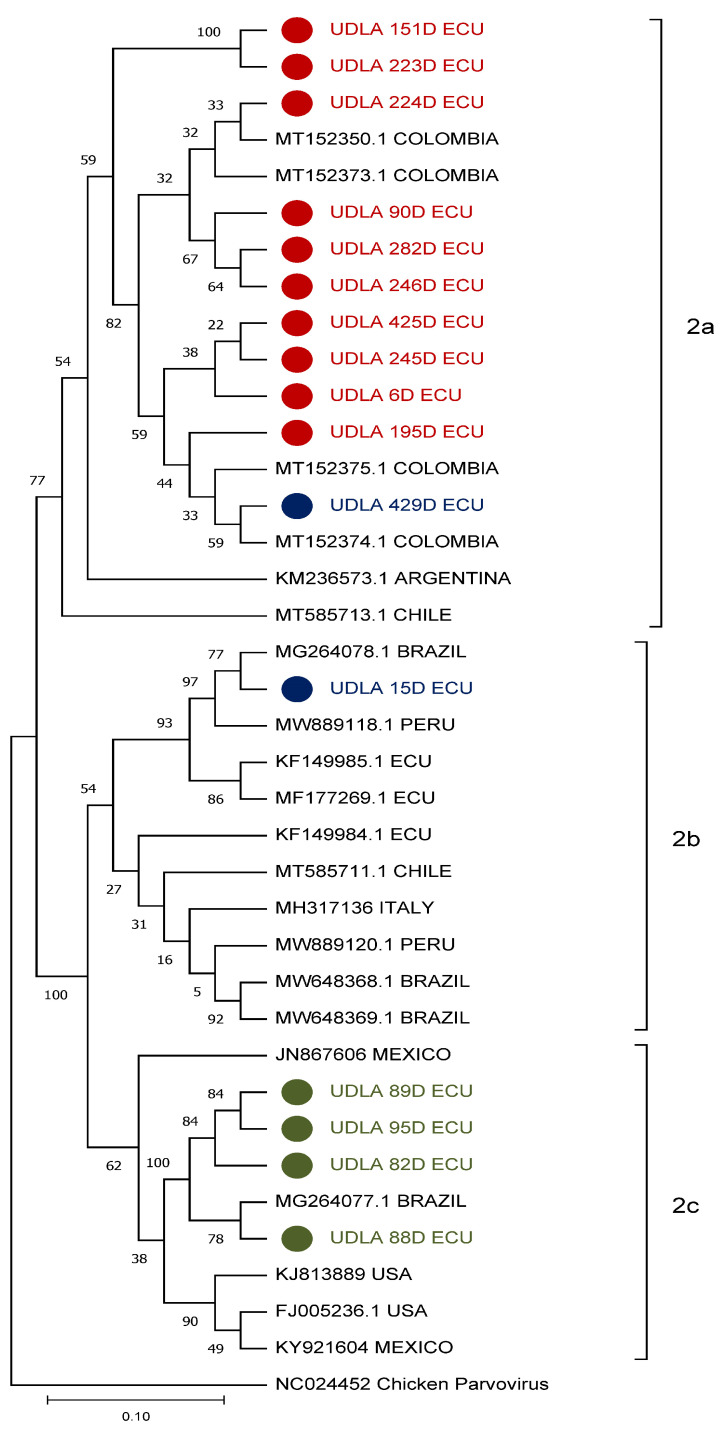
Phylogenetic relationships between the CPV-2 sequences obtained in the present study for the three genotypes and other CPV-2 sequences from the different continents where it is reported at NCBI, based on the VP-1 protein sequence. The sequences were aligned using the CLUSTAL W method in ClustalX2 2.1. The phylogenetic tree was constructed using the MEGA 11 software package. Numbers along the branches refer to bootstrap values for 1000 replicates of chicken parvovirus, which was used as an outgroup. ECU = Ecuador; USA = United States.

All samples were subjected to sequencing; however, only forty-five samples were successful sequenced and genotyped; the remaining CPV-2-positive samples were not sequenced due to the low quality of the extracted DNA. The phylogenetic analysis produced a tree that displays three clades, incorporating CPV-2 sequences generated in this study alongside various sequences obtained from NCBI across different continents for each genotype (Figure 2). Only one of the sequences (UDLA 429D ECU) corresponding to genotype 2b was pooled with genotype 2a.

Comparing the combined CPV-2 sequences of NTs and AAs revealed a high degree of similarity (>97%), with greater variation between variants of AA sequences than between NT sequences (Table 6). Samples 429D and 15D, which correspond to genotypes 2b and 2a, exhibit minimal nucleotide and amino acid similarity to the other documented sequences of the identical genotype, in contrast to the greater similarity of AAs observed in sequences of the same genotype. On the other hand, samples of genotype C exhibit negligible variations in both AAs and NTs.

### 3.5. VP2 Protein Analysis

In the alignment of the amino acid sequences of the VP2 protein were found 23 sites with mutations compared to the reference sequence used, counting the 426th position used to differentiate genotypes 2c and 2b from the CPV-2 and 2a genotypes (Table 7). Genotype 2a sequences show minimal variations from one to two positions except for sample 241D, which shows five mutations between position 139 to 191. Furthermore, mutations were observed at position 297 in multiple samples of genotypes 2b and 2c, which aligns with the area responsible for the formation of the capsid epitope that interacts with the antibody (297-309), thereby enhancing the pathogenic effect of the virus.

## 4. Discussion

During the present study, an epidemiological analysis of the presence of CPV-2 and its variants in Ecuador was obtained using 511 stool samples from dogs with gastroenteric disease, showing the presence of all variants (CPV-2, 2a, 2b, and 2c) in the country. Significantly, this research investigates the utilization of multiplex qPCR-based genotyping as a rapid and cost-effective alternative for sequencing every variant identified in a sample [13,14] and ascertaining the co-occurrence of variants within the same sample. The use of qPCR probes for virus diagnosis has been the gold standard in these procedures for years, due to the speed and specificity of these assays compared to traditional methods [13,17]. The sequencing performed in this study using SANGER technology yielded a variety of CPV-2 sequences from circulating strains in Ecuador as influence material [5]. In the future, the use of NGS techniques is recommended, as they offer advantages in the collection of particular data, faster execution in relation to the number of findings, and increased sequence fidelity [18,19]. Given that the sampling does not follow epidemiological events [20,21,22], prevalence data cannot be determined with fidelity, as in the case of other studies cited; however, this study covers a large number of samples, which serves as supplemental information, improving and increasing the execution of research on these attributes. It is necessary to mention that since all the samples collected correspond to dogs with gastroenteritis, the negative samples for CPV-2 would correspond to other infectious agents such as bacteria, parasites, or other gastroenteric viruses present in canines [20,23,24,25]; therefore, it is necessary to perform consequential studies to identify these agents and possible co-infections among them that generate a more serious presentation of the disease [26,27,28,29].

Previous studies carried out in Japan [9], Egypt [30], and Italy [19] have shown CPV-2 prevalence in 76.7%, 70%, and 54.95% of the samples analyzed, respectively; nevertheless, the present study illustrates a higher percentage of positivity than those previously mentioned, with 78.47% of the samples. On the other hand, studies in Colombia [31] and Peru [32], countries bordering Ecuador, showed the presence of the virus in 51.8% and 57% of the samples analyzed. Although this study indicates a larger number of positive samples compared to all the studies addressed above, it follows a similar trend by revealing positivity in more than 50% of the samples examined. The findings of this study demonstrate that the CPV-2b genotype exhibited the highest positivity rate in qPCR, with 67.11% of the samples testing positive. This finding is consistent with data previously documented in various studies conducted in different countries, which indicate that variants 2b and 2c, in more than 50% of the positive samples, are currently more frequent than the initial strain (CPV-2) [9,22,33]. As previously mentioned, samples obtained from canines from 0 to 12 months showed the highest viral load in the total genotypes (Table 4); this may be related to what was previously described about the lack or disappearance of acquired immunity after the first weeks of life, which increases the susceptibility of individuals to viral infections and hence their mortality [34,35,36]. Although genotype 2b is the genotype most present in the total samples, genotype 2c is shown to produce a higher viral load, as shown in Figure 1 for the 0 to 2 months group; hence, this presents a risk for the canines of this group since, as previously described, genotype CPV-2c is shown to be the cause of the most dangerous version of gastrointestinal disease among the other genotypes and the least susceptible to immunity generated by vaccines due to increases in pathogenicity [22,37,38]. As previously mentioned, genotypes 2a and 2c had the highest viral load in the overall sample (Table 4). Among these genotypes, genotype 2c exhibited the highest average viral copy number within a specific age group (Figure 1). This is consistent with previous findings that suggest a correlation between a high viral load of genotype 2c and increased susceptibility to other infections, including other genotypes of CPV-2. Notably, genotype 2a has been associated with cases of increased mortality [8,22]. Studies in Europe and Asia [9,19,33,39] have emphasized the high distribution and pathogenicity of genotypes 2b and 2c with frequencies of up to 92.4% and 39.57% of positive samples, while studies in Latin American countries have highlighted the presence of genotype 2a with frequencies up to 93.1% [31,32,40]. However, the present study shows a higher prevalence of genotype 2b, corresponding to 85.53% of the positive samples. This could lead to a high risk of mortality in canines infected with this genotype in the country, given its high pathogenicity, as well as a request for further thorough research in neighboring nations where the genotype’s existence has not yet been determined.

The results of the genotype detection analysis, which are presented in Table 3, indicate that distinct combinations of CPV-2 genotypes were co-infected in a single patient, an occurrence that had not been previously documented in the country [18,40]. Thus, the most frequent combination is the one that includes two variants of the virus, 2a and 2b, with 14.48% of the positive samples, which could lead to more aggressive symptoms for the patient, increasing their mortality rate [41,42,43]. Previous studies have documented co-infections involving genotypes 2a and 2c. In such cases, it has been theorized that the coexistence of both virus variants facilitated the expansion of the viral burden within the host organism [22,33]. Conversely, combinations C3 (0.2%), C5 (0.2%), and C12 (0.4%) (Table 3) were observed to a limited degree. When analyzing the samples according to the age of the patients, a range between the ages of 3 and 12 months accounted for 31.31% of the positive samples for all genotypes, with the proportion of positive samples declining significantly with age (Table 3). This could be connected to the decline of acquired immunity during the initial months of a canine’s life, highlighting individuals who are vulnerable to the initial infection with the virus being studied [32,44].

When analyzing the distribution of positive CPV-2 samples across the six provinces sampled in Ecuador, it was discovered that CPV-2 was present in samples from all of the provinces. This finding suggests that the virus may be circulating in these regions as well, in addition to those that have been previously documented [18,41]. When compared to other provinces, Pichincha clearly has predominance, with 44.03% of samples, raising concerns about the large occurrence of this virus. Nevertheless, 58.3% of the total samples came from this single region, with a clear difference between the number of samples from the different regions (Table 6).

Based on statistical analysis comparing the presence of CPV-2 to the patient’s age, it was discovered that the patient’s age (0–12 months) had an impact on the virus’s presence. This finding is consistent with other studies that have shown that the development and growth of the virus affects a canine’s immunity at different stages of its life. Those who are older have likely been exposed to more infectious agents and are therefore better prepared for a viral disease than those who are younger [11,45]. When analyzing the presence of each variant individually and its relationship with age, it was found that the presence of all variants except the CPV-2a variant are influenced by the early age of the patient (0 to 12 months), which indicates that these variants are more likely to be found in the two youngest groups of dogs studied (Table 2), which opens interest in the possibility of a relationship with the presence of this variant and other external factors [36].

The sequencing of the CPV-2-positive samples allowed us to analyze the distribution of the variants present in Ecuador. It should be noted that only 45 of the total number of positive samples could be sequenced, showing that the detection and genotyping methods are efficient regardless of the quality of the DNA due to its low viral load and tendency to fragment over time, while sequencing requires a high viral load and quality [46,47]. The phylogenetic analysis evidenced the presence and distribution of the three CPV-2 variants currently reported in Ecuador and their similarity with variants previously reported in the NCBI. This is except for sample 429D, reported as 2b, but it was mostly similar to the samples corresponding to variant 2a, so it could be due to the presence of co-infections between variants 2a and 2b, given that a similar phenomenon has previously been reported between variants 2a and 2c with recombination (Figure 2) [5,33,48]. When analyzing the similarity between NT and AA sequences, there are clear similarities between the sequences when visualizing NTs, but with variable changes when analyzing AAs, identifying cases in which there are no changes in AAs but there are changes in NTs (Table 6); therefore, the sequences identified show consistency with those previously reported in South America and China [21,32]. An analysis of AA sequences of the VP2 protein shows a series of changes in AA residues ranging from position 28 to position 582. Previously, mutations at positions 440 and 514 have been reported, attributed to methods of differentiation of new unique strains of variants 2c and 2a, respectively [22,40,49]. In particular, the mutation in residue 297 is found in certain sequences of genotypes 2b and 2c, where an asparagine is replaced by an alanine (Table 7), since it has been reported that residues 297-309 form the surface of the capsid protein that binds to the antibody; so, this mutation would cause a decrease in the effectiveness of vaccines based on strains that do not have this mutation [36,50]. In addition, it is likely that a greater number of strains are circulating with this mutation in co-infection; however, only one genotype per sample could be sequenced due to the method used. The presence of co-infection of virus variants at different ages varies drastically in terms of the predominant genotype, so further studies are needed to determine the relationship between the presence of a specific combination and canine mortality [51,52,53]. Thus, it is important to isolate viral genotypes or potential strains with genetic traits of interest and conduct controlled infections in cell lines and subsequently in canines. This will allow for close monitoring of their behavior in controlled environments [54], in addition to the generation and testing of vaccines capable of conferring immunity to canines in each scenario.

## 5. Conclusions

The present study showed for the first time the presence of co-infections between CPV-2 genotypes in dogs of different ages affected with gastroenteric disease in Ecuador, a condition that could influence the severity of the disease. The virus was found to be more prevalent in this region compared to previous studies conducted in similar countries. Furthermore, it was shown that CPV-2 genotypes are present in the canine population of various provinces in Ecuador, including Pichincha, where it was initially reported. The most common genotype observed in the analyzed samples was 2b (Table 2), and mutations were detected in the AAs (Table 7) with the potential to generate pathogenic effects of greater severity.

## Figures and Tables

**Table 1:** Primers used in this study.

Primer	Gene	Assay	Sequences (5′–3′)	Product	Reference
CPV-QF	NS	qPCR	TTCGGTAAACTTAACACCAAC	84 bp	[13]
CPV-QR			CTGTATGTTAATATAGTCACCCA		
CPV-QP			6-FAM-CTGCAATTTCTCTGAGCTTA-MGB		
2161F	VP2	PCR end point	TTGGCGTTACTCACAAAGACGTGC	2662 bp	[5]
4823R			ACCAACCACCCACACCATAACAAC		
CPV-305F		qPCR	CGTTGCCTCAATCTGAAGGAGCTA	85 bp	[14]
CPV-305R			TTGCCCATTTGAGTTACACCACGT		
CPV-2-305P			FAM-ACTCCTATATAACCAAAGTTAGTA-MGB		
CPV-426F		Multiplex qPCR	AGGAAGATATCCAGAAGGAGATTGGA	93 bp	
CPV-426R			CCAATTGGATCTGTTGGTAGCAATACA		
CPV-2a-426P			FAM-CCTGTAACAAATGATAATGTATTGC-MGB		
CPV-2b-426P			VIC-CTTCCTGTAACAGATGATAATGTATT-MGB		
CPV-2c-426P			TAMRA-CTTCCTGTAACAGAAGATAATGTATT-MGB		

**Table 2:** Detection and genotyping of CPV-2 by qPCR according to the age of the patients.

Age (Months)	CPV-2 Detection			Genotyping			
	Negative	Positive	Total	CPV-2	CPV-2a	CPV-2b	CPV-2c
0 to2	30 (27.3%)	85 (21.2%)	115(22.5%)	51 *	47	79 *	49 *
3 to 12	39 (35.5%)	160 (39.9%)	199 (38.9%)	55 *	86	148 *	61 *
13 to 48	34 (30.9%)	104 (25.9%)	138 (27.0%)	19	66	72	36
54 to 96	4 (3.6%)	37 (9.2%)	38 (12.8%)	13	25	31	15
108 to 120	3 (2.7%)	8 (2.0%)	11 (2.2%)	2	7	6	3
132 to 180	0 (0.0%)	7 (1.7%)	7 (1.4%)	4	7	7	3
Total	110 (100.0%)	401 (100.0%)	511 (100.0%)	144	258	343	167

* = statistically significant difference (0.05) among the presence of genotypes of CPV-2 between age groups.

**Table 3:** Analysis of the possible combinations among the reported genotypes of CPV-2 in the samples used in this study.

Combination of Variants					
Combination	CPV-2	CPV-2a	CPV-2b	CPV-2c	TOTAL
C.1	+	+	+	+	35
					
C.2	+	+	+		55
					
C.3	+	+		+	1
					
C.4	+		+	+	36
					
C.5	+	+			1
					
C.6	+		+		10
					
C.7	+			+	4
					
C.8		+	+	+	42
					
C.9		+	+		74
					
C.10		+		+	11
					
C.11			+	+	21
					
C.12	+				2
					
C.14		+			39
					
C.15			+		70
					
C.16				+	17
					

**Table 4:** Gene copies according to age of canines in average and maximum for analysis.

	CG by Genotypes of CPV-2							
Age (Months)	Average				Maximum			
	CPV-2	CPV-2a	CPV-2b	CPV-2c	CPV-2	CPV-2a	CPV-2b	CPV-2c
0 a 2	133 (SD = 1.33 × 10^4^)	5.17 × 10^7^ (SD = 2.07 × 10^8^)	5588 (SD = 7.06 × 10^7^)	1.80 × 10^8^ (SD = 9.01 × 10^8^)	1612	4.73 × 10^10^	1.29 × 10^7^	1.58 × 10^11^
3 a 12	39 (SD = 10)	3036 (SD = 2.10 × 10^8^)	300 (SD = 6.98 × 10^7^)	56 (SD = 9.30 × 10^8^)	39	2.14 × 10^11^	2.43 × 10^10^	2.15 × 10^6^
13 a 48	28 (SD = 17)	115 (SD = 2.07 × 10^8^)	133 (SD = 6.83 × 10^7^)	57 (SD = 8.88 × 10^4^)	52	6.19 × 10^8^	4.80 × 10^7^	8458
54 a 96	34 (SD = 40)	345 (SD = 1.07 × 10^7^)	242 (SD = 4.07 × 10^8^)	50 (SD = 5.02 × 10^8^)	74	4.99 × 10^7^	1.03 × 10^9^	3.01 × 10^9^
108 a 120	4 SD = 0)	173 (SD = 2.70 × 10^8^)	42 (SD = 1165)	48 (SD = 358)	4	1.43 × 10^8^	1186	400
132 a 180	18 (SD = 0)	11203 (SD = 4.01 × 10^8^)	659 (SD = 1.07 × 10^8^)	28 (SD = 50)	89	4.77 × 10^9^	6.38 × 10^8^	72

**Table 5:** The number of samples positive for CPV-2 detection according to the origin of the patients.

		CPV-2	
Province	Negative	Positive	Total
Carchi	1 (0.9%)	64 (16.0%)	65 (12.7%)
Chimborazo	1 (0.9%)	3 (0.7%)	4 (0.8%)
Guayas	8 (7.3%)	42 (10.5%)	50 (9.8%)
Imbabura	6 (5.5%)	22 (5.5%)	28 (5.5%)
* Pichincha	73 (66.4%)	225 (56.1%)	298 (58.3%)
Santo Domingo de los Tsachillas	21 (19.1%)	45 (11.2%)	66 (12.9%)
Total	110 (100.0%)	401 (100.0%)	511 (100.0%)

* = statistically significant difference (0.05) among the presence of CPV-2 in the analyzed samples between provinces where the samples belong to.

**Table 6:** Comparison of the nucleotide and amino acid identities of the CPV-2 sequences detected in Ecuador with other sequences categorized in the same way as found in the phylogenetic tree, including an extract of the sequences of each genotype based on the highest variability.

	Name	1	2	3	4	5	6	7	8	9	10	11	12	13	14	15	16	17	18	19	20	21
		Amino Acid Similarity																				
**1**	MT152350	-	100.0	99.8	98.8	100.0	99.3	98.1	99.1	99.6	99.8	99.6	99.6	98.9	100.0	99.1	99.8	100.0	98.8	99.1	99.1	100.0
**2**	MT152373	99.0	-	99.8	98.8	100.0	99.3	98.1	99.1	99.6	99.8	99.6	99.6	98.9	100.0	99.1	99.8	100.0	98.8	99.1	99.1	100.0
**3**	UDLA 425D	99.0	99.0	-	98.8	99.8	99.3	98.1	99.1	99.9	99.8	99.6	99.6	98.9	99.8	99.1	99.8	99.8	98.8	99.1	99.1	99.8
**4**	UDLA 197D	99.0	99.0	99.0	-	98.8	98.2	96.9	97.9	98.4	98.6	98.6	98.4	97.7	98.8	97.9	99.8	98.8	97.9	97.9	97.9	98.8
**5**	MG264075	99.0	99.0	99.0	99.0	-	99.3	98.1	99.1	99.6	99.8	99.6	99.6	98.9	100.0	99.1	99.8	100.0	98.8	99.1	99.1	100.0
**6**	UDLA 234D	99.0	99.0	99.0	99.0	99.0	-	97.4	98.4	98.2	99.1	99.3	98.9	98.2	99.3	98.4	99.8	99.3	99.1	98.4	98.4	99.3
**7**	MW815499	99.0	99.0	99.0	99.0	99.0	99.0	-	97.2	97.7	97.9	97.7	98.1	97.9	98.1	98.1	99.8	98.1	97.9	98.1	98.1	98.1
**8**	UDLA 151D	99.0	99.0	99.0	99.0	99.0	99.0	99.0	-	98.8	98.9	98.8	98.8	98.1	99.1	98.8	99.8	99.1	98.1	98.8	98.8	99.1
**9**	UDLA 223D	99.0	99.0	99.0	99.0	99.0	99.0	99.0	99.0	-	99.4	99.3	99.6	98.6	99.6	98.8	99.8	99.6	98.6	98.8	98.8	99.6
**10**	MT585713	99.0	99.0	99.0	99.0	99.0	99.0	99.0	99.0	99.0	-	98.8	99.4	99.1	99.8	99.3	99.8	99.8	98.8	99.1	99.1	99.8
**11**	UDLA 429D	98.5	98.5	98.5	98.5	98.5	98.5	98.5	98.5	98.5	98.5	-	99.3	98.9	99.6	99.1	99.8	99.6	98.6	98.9	98.9	99.6
**12**	UDLA 195D	99.0	99.0	99.0	99.0	99.0	99.0	99.0	99.0	99.0	99.0	99.0	-	98.6	99.6	98.8	99.8	99.6	98.6	98.8	98.8	99.6
**13**	UDLA 15D	98.0	98.0	98.0	98.0	98.0	98.0	98.0	98.0	98.0	98.0	98.0	98.0	-	98.9	99.8	99.1	98.9	98.9	99.3	99.1	98.9
**14**	MF177269	98.0	98.0	98.0	98.0	98.0	98.0	98.0	98.0	98.0	98.0	98.0	98.0	99.0	-	99.1	99.8	100.0	98.8	99.1	99.1	100.0
**15**	KF149985	98.0	98.0	98.0	98.0	98.0	98.0	98.0	98.0	98.0	98.0	98.0	98.0	99.0	99.0	-	99.8	99.1	99.1	99.4	99.4	99.1
**16**	MT585711	98.0	98.0	98.0	98.0	98.0	98.0	98.0	98.0	98.0	98.0	98.0	98.0	98.0	98.0	98.0	-	99.8	98.8	99.1	99.1	99.8
**17**	KY921604	98.0	98.0	98.0	98.0	98.0	98.0	98.0	98.0	98.0	98.0	98.0	98.0	98.0	98.0	98.0	99.0	-	98.8	99.1	99.1	100.0
**18**	UDLA 82D	98.0	98.0	98.0	98.0	98.0	98.0	98.0	98.0	98.0	98.0	98.0	98.0	98.0	98.0	98.0	99.0	99.0	-	99.6	99.6	98.8
**19**	MG26077	98.0	98.0	98.0	98.0	98.0	98.0	98.0	98.0	98.0	98.0	98.0	98.0	98.0	98.0	98.0	99.0	99.0	99.0	-	99.9	99.1
**20**	UDLA 88D	98.0	98.0	98.0	98.0	98.0	98.0	98.0	98.0	98.0	98.0	98.0	98.0	98.0	98.0	98.0	99.0	99.0	99.0	99.0	-	99.1
**21**	MZ36281	98.0	98.0	98.0	98.0	98.0	98.0	98.0	98.0	98.0	98.0	98.0	98.0	98.0	98.0	98.0	99.0	99.0	99.0	99.0	99.0	-
		Nucleotide similarity																				
		CPV-2a												CPV-2b				CPV-2c				

**Table 7:** Amino acid mutations in the VP2 protein sequences of CPV-2 analyzed samples.

Sample	Amino Acid Residue Position																									Genotype
	1	20	28	139	144	159	166	174	184	191	199	201	297	311	313	322	324	426	440	514	571	572	577	581	582	
Consensus	M	A	G	V	E	Q	N	M	P	R	P	K	N	D	R	T	I	N	T	S	I	V	Q	R	K	
82D	-	-	-	I	-	-	-	-	-	-	-	-	A	-	-	-	Y	E	-	A	-	-	-	-	-	CPV-2c
88D	-	-	-	-	-	-	-	-	-	-	-	-	A	-	-	-	Y	E	-	A	-	-	-	-	-	
15D	-	-	-	-	-	-	-	-	-	-	-	-	A	-	-	S	Y	D	S	A	-	-	-	-	-	CPV-2b
429D	-	-	-	-	-	-	-	-	-	-	-	-	-	-	-	-	-	D	-	-	-	-	-	-	-	
151D	-	-	-	-	-	-	-	-	-	-	-	-	-	-	-	-	-	-	-	-	-	-	-	-	-	CPV-2a
246D	-	-	-	-	-	H	-	-	A	-	-	-	-	-	-	-	-	-	-	-	-	-	-	-	-	
245D	-	-	E	-	-	-	-	-	-	-	-	-	-	-	-	-	-	-	-	-	F	G	-	S	-	
223D	-	-	-	-	-	-	-	-	-	-	S	-	-	-	-	-	-	-	-	-	-	-	-	-	-	
224D	-	-	-	-	-	-	-	-	-	-	-	-	-	-	-	S	-	-	-	-	-	-	-	-	-	
90D	-	-	-	-	-	-	-	-	-	K	-	E	-	-	-	-	-	-	-	-	-	-	-	-	-	
6D	-	-	-	-	-	-	-	-	-	-	-	E	-	-	-	-	-	-	-	-	-	-	-	-	-	
282D	-	-	-	-	-	-	-	-	-	-	-	-	-	E	k	-	-	-	-	-	-	-	-	-	-	
425D	-	-	-	-	-	-	-	-	-	-	-	-	-	-	-	-	-	-	-	-	-	-	-	-	N	
195D	-	-	-	-	-	-	-	-	-	-	-	-	-	-	-	-	-	-	-	-	-	-	H	-	-	

## Data Availability

This article contains all of the data that were created or analyzed throughout the investigation.

## Supplementary Materials

The following supporting information can be downloaded at: https://www.mdpi.com/article/10.3390/vetsci12010046/s1.
